# Influence of Consumption of Nitrate-rich Beetroot Juice on Lactate Production in Saliva and Oral Biofilm – A Clinical Trial

**DOI:** 10.3290/j.ohpd.b4356487

**Published:** 2023-09-19

**Authors:** Torsten Sterzenbach, Christian Hannig, Susann Hertel

**Affiliations:** a Senior Research Scientist, Clinic of Operative Dentistry, Medical Faculty Carl Gustav Carus, Technische Universität Dresden, Dresden, Germany. Designed the study, drafted the manuscript, performed the experiments, analysed the data, reviewed and edited the manuscript, approved the final version of the manuscript.; b Professor, Clinic of Operative Dentistry, Medical Faculty Carl Gustav Carus, Technische Universität Dresden, Dresden, Germany. Designed the study, reviewed and edited the manuscript, approved the final version of the manuscript.; c Dentist, Clinic of Operative Dentistry, Medical Faculty Carl Gustav Carus, Technische Universität Dresden, Dresden, Germany. Designed the study, drafted the manuscript, performed the experiments, analysed the data, reviewed and edited the manuscript, approved the final version of the manuscript.

**Keywords:** beetroot, caries, nitrate, nitrite

## Abstract

**Purpose:**

Diets rich in nitrates have the potential to prevent oral diseases such as caries or periodontitis. The reduced forms nitrite and nitric oxide have an antibacterial effect against cariogenic bacteria. The effect on bacterial acid production in saliva and oral biofilm is yet unknown. This study investigated the influence of consuming naturally nitrate-rich beetroot juice on bacterial lactate production in saliva and on the pH value of saliva and oral biofilm.

**Materials and Methods:**

In addition to their usual diet, a study group of eight subjects consumed 50 ml of beetroot juice daily for a fortnight. After a two-week break, they rinsed with 0.2% chlorhexidine (CHX) for 14 days as a positive control. Bacterial lactate production was induced by rinsing with 50 ml apple juice and measured at different time points during the study.

**Results:**

After two weeks of daily beetroot-juice consumption, an accumulation of nitrate and nitrite was measured in the saliva. No influence on the bacterial lactate production in saliva or the saliva and plaque pH was found.

**Conclusion:**

Commercially available beetroot juice showed no modulating effects on intraoral bacterial acid production, suggesting no caries-preventive properties under the tested conditions.

Caries is a global disease that affects children and adults alike and leads to irreversible destruction of the tooth structure.^20,35^ The established caries prevention measures in Western countries have led to a significant improvement in dental health over the last fifty years.^22,37^ These include oral health education, oral hygiene instruction and daily use of fluoride toothpaste and mouthrinses. Daily mechanical biofilm removal from the tooth surface is intended to prevent the long-term attachment of cariogenic microorganisms such as *Streptococcus mutans* (*S. mutans*) and *Lactobacillus* spp. and to achieve an antibacterial effect using dental care products, especially fluoride toothpaste. The cariogenic bacterial species produce acids such as lactic and acetic acid through the fermentation of carbohydrates from food. This is especially relevant due to unnaturally high sugar consumption, especially in Western societies.^13^ These acids cause the salivary pH to drop below a critical value of 5.5 and consequently lead to the demineralization of tooth structure.^33^

In view of the possible holistic effects on health, oral health-promoting nutrition is increasingly becoming the focus of dental research.^25,27,38^ Cleave^4^ and Yudkin^39^ hypothesised as early as the 1970s that the overconsumption of fermentable carbohydrates could be assumed to be a causal factor, first for dental and ultimately for systemic diseases such as diabetes, obesity and coronary heart disease.^4,13,39^ For example, milk or plant molecules such as polyphenols have been studied for their effects on oral health.^8,21^ Another plant component with protective effects on general health and possible effects on the oral health is nitrate. Nitrate is an essential nutrient that plants need as a major source of nitrogen, and thus occurs naturally in plants and edible vegetables such as leafy greens, beetroot and radishes in varying concentrations.^12^ When nitrate-containing foods are consumed, nitrate is absorbed from the intestine into the bloodstream via the gastrointestinal tract. If the blood passes the salivary glands, the nitrate (NO_3_^-^) is actively concentrated to high levels (5 to 8 mM) in the saliva.^28^ Oral denitrifying bacteria reduce nitrate to nitrite (NO_2_^-^). The nitrite-rich saliva is swallowed and the nitrite enters the bloodstream via the intestine. In the stomach, blood and tissues, nitrite is further reduced by various processes to nitric oxide (NO), an important cardiometabolic regulator of the human body, including vasodilatation, which lowers blood pressure.^23,28^ Furthermore, NO is also an important inhibitor of endothelial dysfunction.^6^

There is also a direct effect of nitrate when food is chewed or passes through the mouth. Nitrate is reduced through denitrification by oral bacteria to nitrite and further to nitric oxide. Nitrite, as a precursor to NO, is an antimicrobial molecule that restricts the growth of certain bacterial species. Many periodontal pathogenic species are known to be sensitive to NO.^15,16^ Thus, the composition of oral biofilms is affected.^35^ In addition, an acidic decomposition of nitrite to nitric oxide takes place at a pH ≤5. During denitrification and the reduction of nitrite to ammonium, protons are consumed, which increases the local pH.^28^ In-vitro studies have demonstrated that NO_2_^-^ significantly inhibits bacterial acid production by increasing pH through the formation of antimicrobial agents by the formation of antimicrobial NO from NO_2_ under acidic conditions.^26,31^

Hohensinn et al^11^ have shown that NO_3_-rich beetroot juice has a protective effect against caries development by increasing the pH of saliva. The pH value is an important indicator of the number of cariogenic bacteria and a comparatively increased pH value indicates a decrease in the number of acid-producing bacteria. Lactate as a salt of lactic acid is a major metabolic product of oral bacteria.

The aim of our study was to investigate whether a diet rich in nitrate influences salivary nitrate and nitrite concentrations and the influence of a diet rich in nitrates on the lactate production by caries-pathogenic bacteria.

## Materials and Methods

### Study Design

The study consisted of eight healthy subjects. Each study participant signed a written informed consent form. The local Ethics Committee of the Technical University Dresden (EK #147052013) approved the study design.

Participants (n = 8) were asked to drink 50 ml of beetroot juice (Grünland Bio Rote Bete Saft, Lieferello; Kiel, Germany) daily in the evening before dinner for a fortnight. No further changes in dietary habits were to be made. After a two-week break, the subjects were instructed to rinse daily with an 0.2% chlorhexidine (Dynexidin forte 0.2%, Chem. Fabrik Kreussler; Wiesbaden, Germany) rinse after evening dental care for a fortnight.

The beetroot juice used in this study had a nitrate content of 16 mM or 10 g/l NO_3_^-^. This is in line with nitrate contents of other commercially available beetroot juices.^11,41^

Saliva and plaque samples were collected on day 0 (baseline values before first day of beetroot juice consumption), day 15 (after the last day of beetroot juice consumption), day 29 (before the start of daily CHX rinses) and day 43 (after the last day of CHX rinses) in the morning before breakfast.

Each study participant signed a written informed-consent form. The local Ethics Committee of the Technical Universty Dresden (EK #147052013) approved the study design.

### Sample Acquisition

Sampling was conducted in the morning before breakfast. Participants were instructed not to eat or drink (except water) before sample acquisition. Furthermore, participants were asked to omit dental hygiene the morning of sample acquisition.

**Fig 1:**
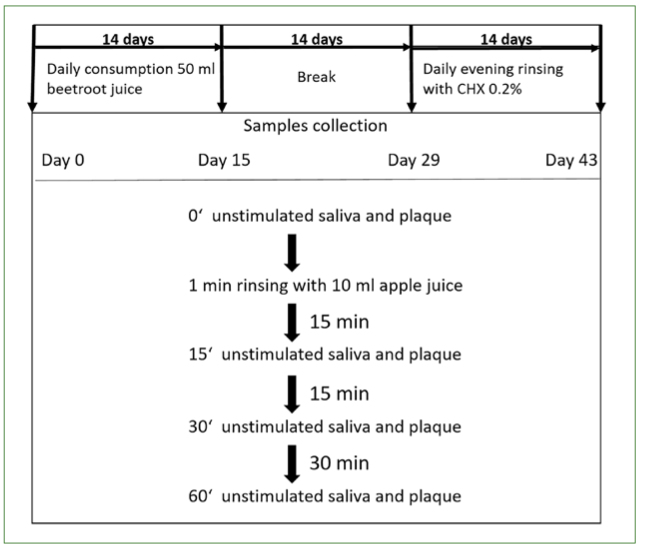
Experimental design: Saliva and plaque samples were obtained on day 0, after 14 days of daily consumption of 50 ml of beetroot juice, after a two-week break and after 14 days of daily rinsing with a CHX solution. At the respective time points, unstimulated saliva and plaque samples were first collected from the experiment (timepoint 0). This was followed by a 1-min rinse with 10 ml apple juice. Further samples were collected again after 15, 30 and 60 min.

A first set of samples (saliva and plaque) was collected at time 0 min. Unstimulated saliva was obtained by spitting into a conical tube. Plaque samples were collected with a microbrush. To induce lactate formation, participants were asked to rinse with 50 ml of apple juice (WeserGold Apfel Direktsaft naturtrüb, riha WeserGold Getränke; Rinteln, Germany) for 1 min. Subsequently, further samples sets (saliva and plaque) were obtained at 15 min, 30 min and 60 min after rinsing with apple juice (Fig 1). The samples were stored at -80°C until further processing.

### Sample Preparation

Plaque samples were dissolved in a salt solution (5.41 mM CaCl_2_, 28.2 mM KCl, 85.6 mM NaCl, 0.2 mM MgSO_4_) at a concentration of 10 mg/ml.

For the lactate assay, both saliva and plaque samples were preprocessed to inactivate proteins and degrade endogenous NaD(P)H dinucleotides. Therefore, samples were incubated at 80°C for 20 min. Then the samples were acidified with 0.6 M HCl at 0.5 times the sample volume (e.g. to 1 ml of sample, 0.5 ml of 0.6 M HCl were added) and incubated for 10 min at room temperature. Finally, samples were neutralised by addition of 0.5 time the sample volume of 1 M Tris base.

### Nitrate und Nitrite Measurements

Concentrations of nitrate and nitrite in samples were determined with a commercial nitrate/nitrite assay kit (Cayman Chemical; Ann Arbor, MI, USA).^10^ The assay is based on the Griess reaction. To determine the nitrite concentration, samples were diluted appropriately in the provided assay buffer. Then 100 µl of sample were mixed with 50 µl each of Griess reagents 1 and 2 and incubated for 10 min. Afterwards, absorbance was measured at 540 nm in a Tecan (Männedorf, Switzerland) reader. In parallel, a standard calibration curve was generated with different concentrations of the provided nitrite standard. Nitrite concentration of the salivary samples was then calculated from the standard curve. To determine the combined nitrate and nitrite concentrations, samples were preincubated with the provided nitrate reductase mix for 3 h. In this process, nitrate in the samples was reduced to nitrite. Afterwards, combined nitrate and nitrite concentration in the saliva samples was determined as described for determining nitrite concentration. To determine nitrate, the concentration of nitrite was subtracted from the concentration of combined nitrate and nitrite.

### Determination of Lactate

Lactate concentration was determined with the Lactate-Glo assay by Promega (Madison, WI, USA).^5^ Saliva and plaque samples were pretreated as described above.

For the assay, 10 µl of sample were mixed with 30 µl of ddH_2_O (double-distillied water) and 10 µl of lactate detection reagent according to the manufacturer’s instructions. If necessary, samples were prediluted in ddH_2_O to be within the linear range of the assay. In parallel, suitable concentrations of lactate were used to generate a calibration curve. Luminescence was then recorded in a Tecan plate reader at 5-min intervals until a plateau was reached. Concentrations of lactate were calculated from the calibration curve obtained with defined lactate concentrations.

### Glucose Assay

Concentrations of glucose were measured with the D-glucose/D-fructose UV test by R-Biopharm (Pfungstadt, Germany). Therefore, 100 µl of sample were mixed with 100 µl ddH_2_O and 100 µl of solution 1 provided by the manufacturer. Then the extinction was measured at 340 nm in a Tecan reader (E1), after which 2 µl of solution 2 containing hexokinase and glucose-6-phosphate-dehydrogenase were added. The extinction at 340 nm was measured until maximal extinction was reached (E2). Concentrations of glucose were then calculated as described by the manufacturer. As a control, defined concentrations of glucose were measured in parallel.

### Statistical Analysis

Statistical analyses was conducted with GraphPad Prism (Boston, MA, USA) using a two-tailed t-test.

## Results

### Study Participants

Eight healthy subjects (6 female, 2 male) aged between 23-46 years voluntarily participated in this longitudinal intervention study. The subjects did not take any medication regularly.

### Consumption of Beetroot Juice Increases the Concentration of Nitrite in Saliva

**Fig 2:**
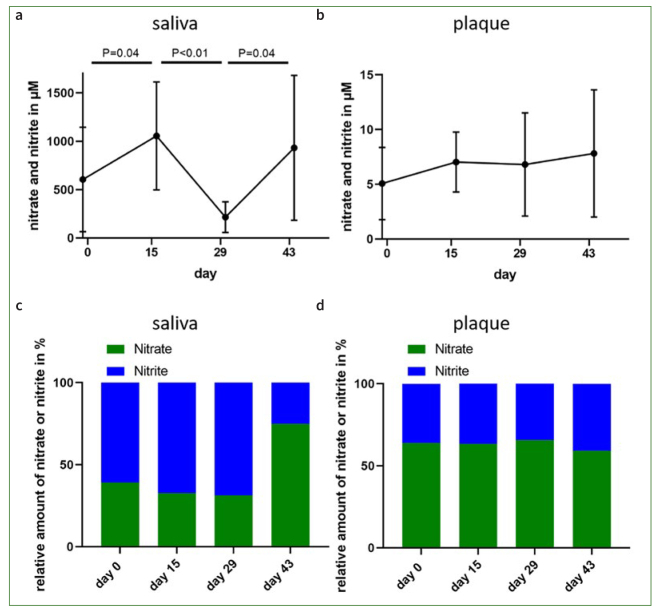
Temporal changes in the concentrations of nitrate and nitrite in saliva and plaque before (day 0) and after two weeks of daily consumption of beetroot juice (day 15) as well as after discontinuation of the juice (day 29) and after 14 days of rinsing with CHX 0.2% (day 43). The concentrations of nitrate and nitrite were measured in saliva samples (a) or from plaque samples (b). Relative distributions of nitrate and nitrite in saliva- (c) or plaque samples(d) obtained on the different test days. Data are given as mean values ± SEM, n = 8 subjects. Statistical significance was determined using the t-test.

The participants were asked to consume a commercially available brand of beetroot juice for 14 days. It was observed that statistically significantly higher concentrations of nitrate and nitrite were measured in saliva after consuming beetroot juice for 2 weeks (Fig 2a and Table 1).

**Table 1:** Overview of mean (± SD) salivary nitrate and nitrite concentrations during the trial, n = 8 subjects

	Salivary nitrate and nitrite in µM							
	Day 0		Day 15		Day 29		Day 43	
	Mean	SD	Mean	SD	Mean	SD	Mean	SD
Nitrate	234.4	194.6	353.3	341.6	82.4	111.8	713.4	569.7
Nitrite	370.1	347.5	701.2	510.5	133.5	76.7	218.6	161.7
Nitrate + nitrite	604.5	504.6	1054.5	522.4	214.6	148.3	932.0	702.1

As expected, concentrations of nitrogen compounds decreased again after cessation of beetroot juice consumption (Fig 2a, day 29). Surprisingly, concentrations were on average below levels measured on day 0. At day 43 after 2 weeks of daily rinsing with 0.2% CHX, overall nitrate and nitrite levels reached levels comparable to initial concentrations on day 0. The concentration of nitrate/nitrite in the plaque did not change statistically significantly throughout the study period (Fig 2b and Table 2). The examination of the saliva and plaque samples showed a high interindividual variability with regard to the results (Figs 2 a/b).

**Table 2:** Overview of mean (± SD) nitrate and nitrite concentrations in plaque samples during the trial, n = 8 subjects

	Plaque nitrate and nitrite in µM							
	Day 0		Day 15		Day 29		Day 43	
	Mean	SD	Mean	SD	Mean	SD	Mean	SD
Nitrate	3.98	3.13	5.70	4.38	5.27	4.82	6.70	6.82
Nitrite	1.86	2.12	3.17	4.15	3.36	4.35	2.50	3.13
Nitrate + nitrite	5.07	3.09	7.04	2.56	6.81	4.41	7.81	5.44

The ratio between nitrate and nitrite remained rather constant for samples obtained on days 0, 15 and 29. This suggests that consumption of beetroot juice does not influence the balance between nitrate and nitrite obtained by deniftrification by the oral microbiota. However, daily rinsing with CHX resulted in an increased salivary amount of nitrate compared to nitrite, while the ratio in the plaque samples remained unaffected (Figs 2c and 2d).

### Lactate Production Did Not Change with Increased Concentration of Nitrogen Compounds

At the four sample-acquisition time points, participants were asked to rinse with apple juice for 1 min. Additional salivary samples were obtained 15, 30 and 60 min after rinsing with apple juice. The sugar content of apple juice is mainly composed of fructose with lower amounts of sucrose and glucose.^19^

**Fig 3:**
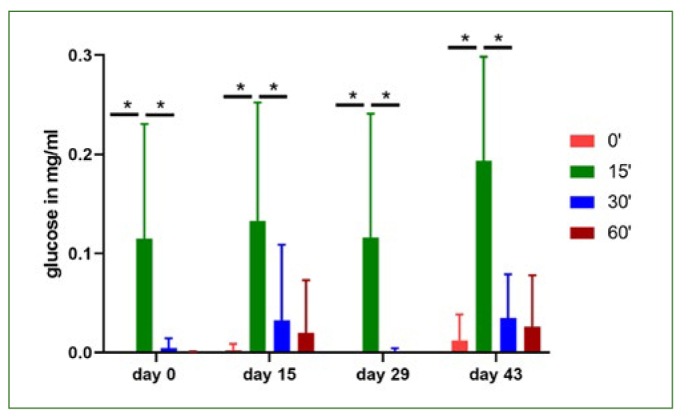
Concentrations of glucose in saliva. The glucose concentrations in saliva were measured using samples obtained on the corresponding test days in the morning, fasting before the test (timepoint 0), and 15, 30 and 60 min after a 1-min rinse with apple juice. Data are given as means ± SEM, n = 8 subjects. Statistical significance was determined using the t-test. *p < 0.05.

**Fig 4:**
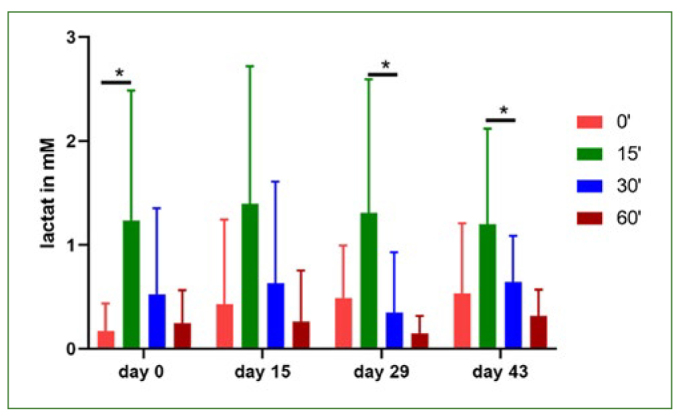
Concentrations of lactate in saliva. The lactate concentrations in saliva were measured in samples taken on the corresponding test days fasting before the test (timepoint 0) as well as 15, 30 and 60 min after a 1-min rinse with apple juice. Data are given as means ± SEM, n = 8. Statistical significance was determined using the t-test. *p < 0.05.

First, concentrations of glucose in saliva before rinsing with apple juice was measured. For baseline measurements, the experiments took place in the morning and participants were asked not to eat or drink anything except water on the morning of sample acquisition. As expected, concentrations of glucose peaked 15 min after rinsing with apple juice. After 30 min and 60 min, glucose was still detectable at low levels (Fig 3 and Table 3). Next, lactate production in saliva was measured after rinsing with apple juice. As expected, an increase in lactate concentration was detected 15 min after rinsing compared to the baseline levels in samples obtained directly before rinsing with apple juice. Lactate concentrations dropped with time and reached baseline levels 60 min after the rinse with apple juice. However, no differences were observed between the four different days of sample acquisition. No significant differences were observed both in the baseline levels and lactate production after rinsing with apple juice both after consumption of beetroot juice and after rinsing with chlorhexidine (Fig 4 and Table 4).

**Table 3:** Overview of mean (± SD) salivary glucose concentrations during the trial, n = 8 subjects

Time after rinsing with apple juice in min	Glucose in mg/ml							
	Day 0		Day 15		Day 29		Day 43	
	Mean	SD	Mean	SD	Mean	SD	Mean	SD
0	0.000	0.000	0.000	0.000	0.000	0.000	0.012	0.025
15	0.115	0.108	0.133	0.112	0.116	0.118	0.193	0.098
30	0.005	0.010	0.033	0.071	0.001	0.003	0.035	0.041
60	0.000	0.000	0.020	0.050	0.000	0.000	0.026	0.050

**Table 4:** Overview of mean (± SD) salivary lactate concentrations during the trial, n = 8 subjects

Time after rinsing with apple juice [min]	Lactate in mM							
	Day 0		Day 15		Day 29		Day 43	
	Mean	±	Mean	±	Mean	±	Mean	±
0	0.172	0.247	0.428	0.765	0.492	0.469	0.536	0.627
15	1.238	1.168	1.396	1.238	1.308	1.202	1.198	0.862
30	0.526	0.772	0.633	0.913	0.350	0.541	0.647	0.413
60	0.250	0.294	0.266	0.456	0.152	0.154	0.318	0.238

**Fig 5:**
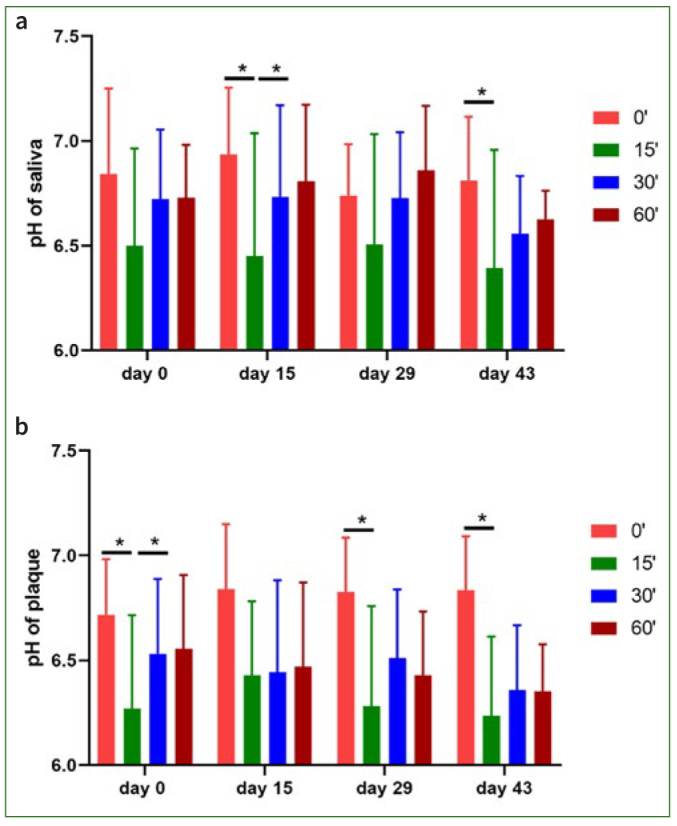
Temporal changes of pH in saliva (a) and plaque (b) during 14 days of consumption of beetroot juice and after discontinuation as well as after the two-week rinse with CHX. The pH measurements were taken on the respective test days before breakfast (timepoint 0) and 15, 30 and 60 min after a 1-min rinse with apple juice. Data are given as mean values ± SEM, n = 8 subjects. Statistical significance was determined using the t-test. *p < 0.05.

The pH value was measured in the saliva and plaque samples. No significant differences were found between the different collection times for the initial samples before rinsing with apple juice. As expected, the pH of the saliva and plaque samples decreased 15 min after rinsing with apple juice. At 30 min and 60 min after rinsing, the pH value increased again. However, the pH changes due to rinsing with apple juice were comparable at all four sampling times both for saliva and plaque samples (Fig 5 and Table 5).

**Table 5:** Overview of mean (± SD) salivary and plaque pH during the trial, n = 8 subjects

Time after rinsing with apple juice in min	pH values in saliva							
	Day 0		Day 15		Day 29		Day 43	
	Mean	SD	Mean	SD	Mean	SD	Mean	SD
0	6.84	0.38	6.94	0.30	6.74	0.23	6.81	0.29
15	6.50	0.43	6.45	0.55	6.51	0.49	6.39	0.53
30	6.72	0.31	6.73	0.41	6.73	0.29	6.56	0.26
60	6.73	0.24	6.81	0.34	6.86	0.29	6.63	0.13
Time after rinsing with apple juice in min	pH values in plaque							
	Day 0		Day 15		Day 29		Day 43	
	Mean	SD	Mean	SD	Mean	SD	Mean	SD
0	6.72	0.25	6.84	0.29	6.83	0.24	6.84	0.24
15	6.27	0.42	6.43	0.33	6.28	0.45	6.24	0.35
30	6.53	0.33	6.44	0.41	6.51	0.31	6.36	0.29
60	6.55	0.33	6.47	0.38	6.43	0.28	6.35	0.21

## Discussion

The aim of the study was to investigate the impact of a nitrate-containing food on the accumulation of nitrate and nitrite in the oral cavity. For this purpose, the participating subjects consumed 50 ml of naturally nitrate-containing beetroot juice daily for 14 days. Saliva and plaque samples were taken before starting the diet (day 0), at the end (day 15) and 14 days after daily consumption of beetroot juice (day 29). Carbohydrates such as glucose can be fermented to lactate by cariogenic bacteria. Hence, another aim was to investigate whether changes in the nitrate or nitrite concentration in saliva have an influence on bacterial lactate production. Therefore, lactate production was induced on the respective test days by rinsing with apple juice. As a control, at the end of the 28-day course, subjects were asked to rinse daily with 0.2% chlorhexidine (CHX) for a further two weeks. It was hypothesised that by reducing the total bacterial count, reduced lactate production in saliva could be achieved.

This study showed that statistically significantly higher concentrations of nitrate and nitrite were measured in saliva after daily consumption of beetroot juice over a period of two weeks. This confirms previous studies on the accumulation of nitrate or nitrite in saliva after the consumption of nitrate-containing foods.^3,11,24^ The effect was comparable to the results of Hohensinn et al,^11^ although for comparison we used a commercially available beetroot juice with a statistically significantly higher nitrate concentration (mixed juice with other vegetable and fruit juices) over the same period of time.^11^ However, an influence on the nitrate/nitrite concentration in the oral biofilm (plaque) could not be determined. This reconfirms the uniformity and selectivity of biofilm formation on dental hard tissues as well as the resilience of the oral microbiome postulated in numerous previous publications on bioadhesion processes.^7,9,31,36^

Despite the increased nitrate and nitrite concentrations in saliva, no effects on bacterial lactate production were found. Previous studies suggested that the production of acids from carbohydrates might be reduced by the increased presence of nitrate and nitrite in saliva.^11^ Rosier et al^28^ postulated two possible hypotheses for this: first, that nitrate could limit acidification by consuming lactic acid, since bacteria can use lactic acid as a carbon source and electron donor during the metabolic reduction of nitrate and nitrite;^28,29^ second, the antimicrobial activity of nitric oxide, which is produced during denitrification and could influence oral communities, is described as a cause of reduced lactate production.^28,35^ It is conceivable that the concentration of nitrate accumulation in saliva is too low to cause substantial antibacterial effects from the denitrification products nitrite and nitric oxide. According to Radcliff et al,^26^ nitrite has a highly statistically significant bactericidal effect on *S. mutans* at concentrations of 20 and 200 mM. In our study, these values were not reached. This might also explain why no influence on the pH in saliva and plaque could be detected. In future studies, higher doses of beetroot juice could be used to possibly obtain higher concentrations of nitrite or nitric oxide.

The two-week rinse with 0.2% CHX was assumed to be a positive control, as the reduction in the total number of bacteria also reduced lactate production in saliva. However, contrary to our assumption, it was found that higher glucose and lactate levels tended to be measured in saliva after CHX application compared to day 0, and no effect on saliva and plaque pH could be detected.

This correlation was also reported by Bescos et al.^2^ In their study, the effect of a one-week rinse with CHX on various salivary markers was investigated in 36 subjects. They found that after the application of CHX, salivary pH was lower and lactate and glucose concentrations were higher, which is confirmed by our results.^2^ Although it has long been proven and is not questioned here that mouthwashes with CHX are effective in reducing plaque accumulation and gingivitis,^1,14^ the impact on the oral microbiome can now be considered in a more nuanced way thanks to modern genome sequencing. The antibacterial properties of CHX lead to reduced oral microbial diversity.^2^ The research by Bescos et al^2^ showed that this results in an increase in the Firmicutes group, which mainly consists of genera such as *Streptococcus*. These lactic acid-producing bacteria have a selection advantage at low pH and thus even increase lactate production. At the same time, there is a statistically significant decrease in *Bacterioides* and *Veillonella* after CHX application, both of which are important for maintaining intraoral acid-base balance.^2^ Taking this into account, and in conjunction with the results of our study, it strongly suggests that CHX promotes salivary acidification and thus increases the risk of caries to a relevant extent with prolonged use.

Our study resulted in an increased amount of salivary nitrate compared to nitrite, ie, the ratio between nitrate and nitrite shifted in favour of nitrate. This can be attributed to the reduction in the total number of bacteria and in particular to the reduction of nitrate-metabolising species of the genera *Veillonella* and *Actinomyces*.^2^ Kapil et al^17^ also showed that rinsing with CHX for one week reduced oral nitrite production by 90% and consequently plasma nitrite levels by 25%, which also influenced the naturally regulating vasodilatory effect on the blood pressure system. There are studies suggesting that prolonged CHX applications lead to higher blood pressure in both healthy^17^ and hypertensive subjects.^18^ However, no effect on the cardiovascular system is expected after 3-7 days.^33^

It should be noted that this study also has some limitations. Our participants were not required to follow a specific diet but to continue their regular diet. In this way, secondary effects caused by an abrupt change in diet of the participants could be avoided. However, this could also mask the effects caused by the beetroot juice or apple juice challenge, especially if participants regularly consume a nitrate-rich or carbohydrate-rich diet. Furthermore, only one defined dose of beetroot juice or apple juice for the lactate challenge were used. Future follow-up studies could modulate the dose of both beetroot juice as well as apple juice. In addition, longer times for the dietary regime would be possible.

## Conclusion

Despite the limitations of our study, it can be concluded that regular consumption of commercial beetroot juice does alter the nitrate and nitrite concentrations in saliva, but a caries-preventive effect could not be demonstrated. Furthermore, the sugar content of beetroot juice deserves critical mention. Even with a potentially antibacterial nitrite effect, metabolisation of the low-molecular carbohydrates takes place in the plaque or existing carious lesions. The overall health effect of a diet rich in nitrates should not be questioned here, but the caries-preventive potential could not be confirmed under the circumstances tested.
